# Pseudoaneurysm inside of a true aneurysm

**DOI:** 10.1186/1749-8090-8-97

**Published:** 2013-04-17

**Authors:** Pedro Sousa, Walter Santos, Pedro Cordeiro, Salomé Pereira, Rui Ferrinha, Victor Brandão, Manuel P Magalhães, Ilídio Jesus

**Affiliations:** 1 Cardiology Department, Faro Hospital, Faro, Portugal; 2 Cardiothoracic Surgery Department, Cruz Vermelha Portuguesa Hospital, Lisbon, Portugal

**Keywords:** Pseudoaneurym, True aneurysm, Echocardiography

## Abstract

Left ventricular pseudoaneurysms and true aneurysms are two possible complications of myocardial infarction. However, while pseudoaneuryms require urgent surgical resection, true aneuryms can often be managed medically, making imperative an accurate diagnosis.

The authors describe a case of a delayed rupture of a true aneurysm that was contained and gave rise to a pseudoaneurysm inside of a true aneurysm. The echocardiography allowed the differential diagnosis for a timely surgical intervention which resulted in the patient’s full recovery.

## Background

Left ventricular (LV) aneurysms are a possible consequence of extensive myocardial necrosis that occurs more often as a result of an anterior MI.

Rupture of the LV free wall is a rare but catastrophic complication of an acute MI [1]. Rarely, rupture is contained by the overlying pericardium resulting in a pseudoaneurysm [1,2]. The diagnosis of LV pseudoaneurysm can be challenging, because patients are often asymptomatic or present nonspecific symptoms [1-3]. For this reason, pseudoaneurysm is frequently an incidental finding on imaging tests [1,4].

While pseudoaneurysms require urgent surgical resection because of the likehood of rupture, true aneurysms can often be managed medically. This difference makes imperative an accurate diagnosis [1,2,4].

Echocardiography proves to be valuable in this differential diagnosis. An echocardiographic series reported that in pseudoaneurysms the ratio of the maximum diameter of the neck to the maximum internal diameter of the cavity was between 0.25 and 0.50 [1,3]. Conversely, this ratio is > 0.50 in true LV aneurysms. Pseudoaneurysms are also more often located in the posterior or inferior wall [1,3,4]. One possible explanation is that rupture of the anterior wall usually results in immediate death, in contrast with rupture of the posterior wall [1].

The authors describe a case of a delayed rupture of a true aneurysm that was contained by the pericardium, which prevented the patient death and gave rise to a pseudoaneurysm inside of a true aneurysm. Echocardiography made the diagnosis which was later confirmed by the surgical report.

## Case presentation

We report a case of a 57-year-old man, caucasian, with a history of hypertension, dyslipidemia and former smoking, that suffered 13 years ago an acute antero-lateral myocardial infarction (MI) and was then submitted to angioplasty with stents implantation in the left anterior descending (LAD) and circumflex arteries. In the follow-up he was informed that he had developed a left ventricular (LV) aneurysm.

He remained asymptomatic until 2 years ago, when he was brought to the Emergency Department with a syncopal episode. The ECG on admission revealed the presence of sinus rhythm with Q waves, ST segment elevation and negative T waves in the anterior and lateral leads, which were findings that were already known for previous ECG after MI. The biomarkers of myocardial necrosis were elevated with a peak troponin I of 3,2 μg/L. He was then admitted to the Cardiology Department with the diagnosis of non-ST segment elevation acute MI.

**Figure 1:**
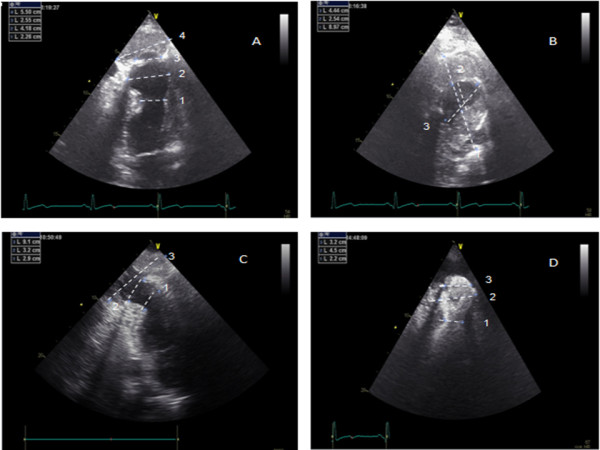
**Transthoracic echocardiogram revealing the pseudoaneurysm with a large thrombus inside. A**- Four chamber view (1-aneurysm neck; 2-aneurysm maximum diameter; 3- pseudoaneurysm neck; 4- pseudoaneurysm maximum diameter), **B**- Parasternal short axis view at apical level (1- pseudoaneurysm maximum diameter; 2 and 3- pseudoaneurysm neck) **C**- Two chambers view in systole with the maximum diameter of the neck [2] and the maximum internal diameter of the pseudoaneurysm [3]. **D**- Four chamber view using contrast (1-aneurysm neck; 2- aneurysm maximum diameter; 3- pseudoaneurysm neck).

The transthoracic echocardiography (Figure 1) revealed a moderate LV systolic dysfunction with hypokinesia of the anterior and inferior walls and septum and an apical LV aneurysm. Surprisingly, the apical aneurysm contour exhibited an abrupt discontinuity that gave access to another cavity filled with an echodense material. The relationship between the maximum diameter of the neck and the maximum internal diameter of this cavity was inferior to 0.5, suggesting that this cavity was a pseudoaneurysm with a large thrombus inside.

**Figure 2:**
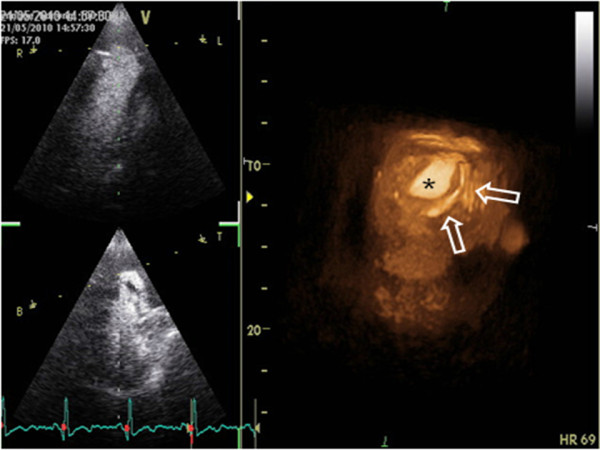
3-D echocardiography with constrast, revealing calcification of the pericardium (arrows) and echo-contrast filling the pseudoaneurysm neck (*).

3-D echocardiography showed calcification of the pericardium and echo-contrast filling out the pseudoaneurysm neck (Figure 2).

**Figure 3:**
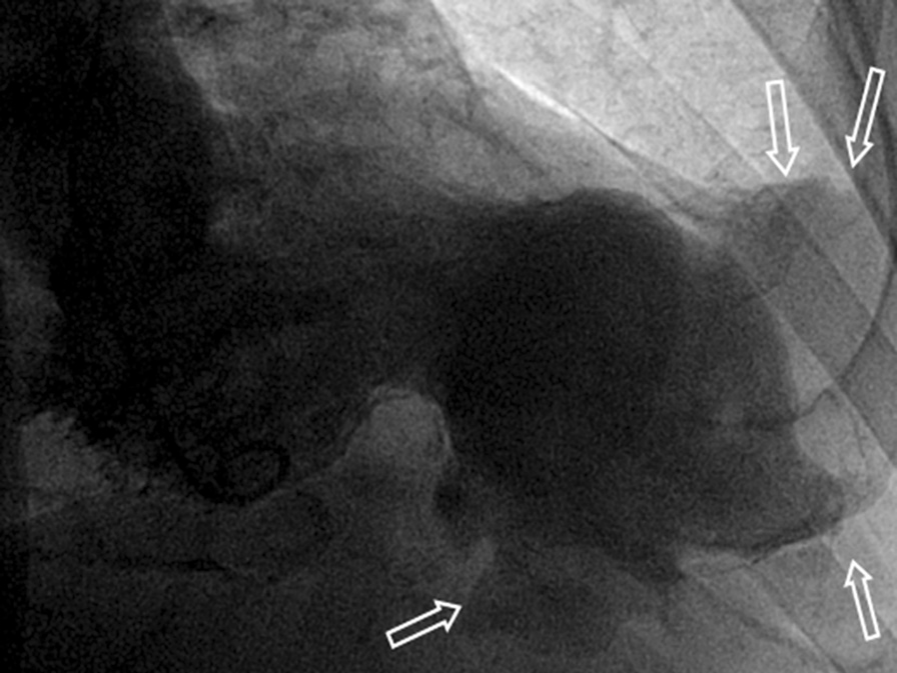
Left ventriculography revealed a pseudoaneurysm with a thrombus.

The coronary angiography revealed a sub-occlusive stenosis of the stent in the LAD and the left ventriculography demonstrated an apical aneurysm surrounded by another giant apical cavity suggestive of a pseudoaneurysm that seemed to be carpeted by a thrombus (Figure 3).

Patient underwent coronary artery bypass surgery and resection of the pseudoaneurysm –externally the apex region was fully adherent to the pericardium with areas of calcification on the “wall” making the dissection impossible in this area. A circular incision was made around the perimeter of the apex and it was evident in this cutting zone that there was no muscle tissue and that the residual wall was formed by thickened pericardium and organized thrombus.

**Figure 4:**
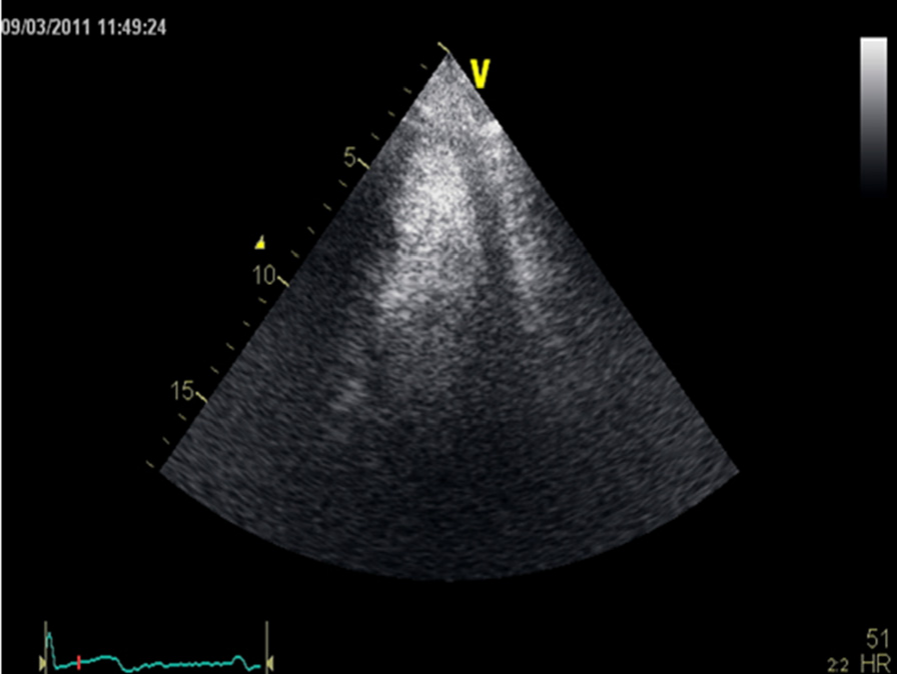
Constrast echocardiography performed one year after surgery showed a preserved LV systolic function, without evidence of a pseudoaneurysm.

One year after the surgery, the patient remained asymptomatic and without any image suggestive of pseudoaneurysm in the apical region of the left ventricle (Figure 4).

## Conclusions

This clinical case is interesting in several aspects. First, it reports a delayed rupture of a true aneurysm that probably occurred 11 years after the aneurysm formation. This is a very rare event, because the potential for rupture of a true aneurysm is higher in the 2–3 weeks after an acute MI.

Second, this is a rare case of a rupture of a true aneurysm that was contained by the thrombus formation and the pericardial adherences secondary to the previous MI. This is an impressive event because it prevented the patient death and gave rise to a pseudoaneurysm inside of a true aneurysm. This pseudoaneurysm is consequently located in the apical region, unlike the majority of cases of pseudoaneurysms. Impressive images of this rare echocardiographic finding are provided in this case report.

Finally, it highlights the importance of standard, contrast and 3-D echocardiography not only for the detection of LV pseudoaneurysms, but also for its differential diagnosis. Because pseudoaneurysms have a high probability of rupture and death, not only during the early stages of its development but also during the established fibrous stage [5], the patient was quickly sent to surgery with no time to perform MRI. The preoperative diagnosis of the pseudoaneurysm was then essentially based on echocardiography and the definitive diagnosis was only finally established by the surgical report.

## Consent

Written informed consent was obtained from the patient for publication of this case report and any accompanying images. A copy of the written consent is available for review by the Editor-in-Chief of this journal.

## Abbreviations

LV: Left ventricular; MI: Myocardial infarction; LAD: Left anterior descending; ECG: Electrocardiogram.

## Competing interests

The authors declare that they have no competing interests.

## Authors’ contribution

MPM performed the surgery and was responsible for the surgical discussion. PC and RF evaluated the echocardiographic images. VB performed the coronary angiography and the left ventriculography. WS, SP and IJ were major contributors in writing the manuscript. All authors read and approved the final manuscript.
